# Replication fork stalling in late S-phase elicits nascent strand degradation by DNA mismatch repair

**DOI:** 10.1093/nar/gkae721

**Published:** 2024-08-24

**Authors:** Erica Colicino-Murbach, Caitlin Hathaway, Huzefa Dungrawala

**Affiliations:** Department of Molecular Biosciences, University of South Florida, Tampa, FL, USA; Department of Molecular Biosciences, University of South Florida, Tampa, FL, USA; Department of Molecular Biosciences, University of South Florida, Tampa, FL, USA

## Abstract

Eukaryotic chromosomal replication occurs in a segmented, temporal manner wherein open euchromatin and compact heterochromatin replicate during early and late S-phase respectively. Using single molecule DNA fiber analyses coupled with cell synchronization, we find that newly synthesized strands remain stable at perturbed forks in early S-phase. Unexpectedly, stalled forks are susceptible to nucleolytic digestion during late replication resulting in defective fork restart. This inherent vulnerability to nascent strand degradation is dependent on fork reversal enzymes and resection nucleases MRE11, DNA2 and EXO1. Inducing chromatin compaction elicits digestion of nascent DNA in response to fork stalling due to reduced association of RAD51 with nascent DNA. Furthermore, RAD51 occupancy at stalled forks in late S-phase is diminished indicating that densely packed chromatin limits RAD51 accessibility to mediate replication fork protection. Genetic analyses reveal that susceptibility of late replicating forks to nascent DNA digestion is dependent on EXO1 via DNA mismatch repair (MMR) and that the BRCA2-mediated replication fork protection blocks MMR from degrading nascent DNA. Overall, our findings illustrate differential regulation of fork protection between early and late replication and demonstrate nascent strand degradation as a critical determinant of heterochromatin instability in response to replication stress.

## Graphical Abstract

**Graphical Abstract:**
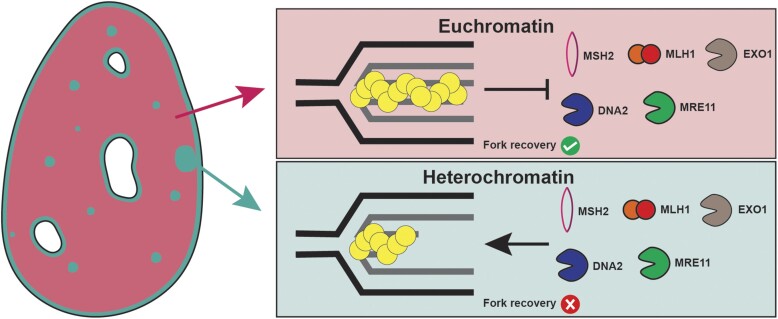

## Introduction

Chromosomes are copied accurately and completely during each round of DNA synthesis. Post DNA replication, eukaryotic genome is rapidly assembled around histone octamers to form nucleosomes and re-arranged into higher order chromatin structures (1). The chromosomal material is spatially segregated into two distinct compartments—euchromatin (EC) and heterochromatin (HC) (2,3). HC is further characterized into developmentally controlled facultative heterochromatin and constitutive heterochromatin which is invariably present in all cell types. While EC is considered to be more open, transcriptionally active and readily prone to transcription-replication conflicts, HC is generally highly compact, relatively gene poor, transcriptionally suppressed and enriched with epigenetic modifications associated with repressive histone marks (4). Constitutive HC consists of telomeric and pericentromeric regions, while repetitive elements in centromere and non-repetitive elements in Lamin Associated Domains (LADs) also share repressive signature of HC (5). Histone modifications and resulting protein associations are highly varied between EC and HC making the two chromosomal territories functionally and structurally separable (6). Cells establish a temporal order of DNA replication early in G1 phase called replication timing program to duplicate euchromatic and heterochromatic DNA in distinct times during S-phase (7). In general, EC replication occurs early in S-phase and mainly appears in the nucleoplasm whereas replication of the condensed chromatin is observed at nuclear periphery, nucleolar periphery and chromocenters in mid to late S-phase (8,9). As such, the process of DNA replication is highly orchestrated and regulated to ensure faithful inheritance and propagation of both genetic and epigenetic information.

A constant threat to genome maintenance is exposure to both endogenous and exogenous genotoxic stress. The highly compact nature and increased propensity to illegitimate recombination of repetitive sequences in heterochromatic DNA represents a unique and challenging environment for DNA damage sensing, signaling and repair (3,5,10,11). Several studies demonstrate differential degree in DNA damage and subsequent repair kinetics between EC and HC in resolution of UV-induced CPD photoproducts (12,13) and repair of double strand breaks (DSBs) by homologous recombination (HR) (5,14). In flies, the early steps of sensing DSBs, checkpoint activation and DNA processing by resection occur within HP1α-marked HC domains with similar efficiency as euchromatic breaks. However, a network of proteins occlude RAD51 from DNA damage sites in HC to deter erroneous recombination between repetitive DNA (15,16). Subsequently, breaks are relocalized to the nuclear periphery by nuclear actins and myosins in order to access RAD51 for completion of HR (17). Relocalization of breaks and exclusive RAD51-association with periphery was also observed in response to CRISPR/Cas9-induced breaks in mouse and human cells (18,19) demonstrating that the relocalization pathways and spatial regulation of HR-dependent DSB repair is highly conserved across species. Furthermore, DSB repair in nuclear lamina is restrictive to HR and predominantly relies on end joining repair mechanisms (20) demonstrating a role of HC superstructure in negatively regulating HR (19). Genome-wide studies in cancers have reported higher mutation rates in heterochromatic DNA (21–24) further illustrating a strong link between chromatin organization, differential DNA repair and genome stability.

Bulk analyses of DNA replication using cellular and DNA fiber assays with thymidine analogs indicate that elongation rates gradually increase from early to late S-phase (25–28), with one study demonstrating uniform elongation rates across S-phase in multiple cell types (29). These observations imply that the condensed state of chromatin in mid to late S-phase minimally restricts fork progression. In addition to compaction state, HC is enriched with difficult-to-replicate regions such as telomeres, transposons and satellite repeat sequences in pericentric DNA. Several common fragile sites, that share a common feature of late replication (30,31), are characterized by histone hypoacetylation (32). These regions act as natural obstacles to moving replication forks and are exquisitely sensitive to replication stress as exposure to mild doses of DNA replication impediments generate chromosomal breaks and segregation errors. The underlying factors contributing to DNA breakage in these regions include fork stalling by secondary structures (33), paucity of replication origins (34–36) and topological constraints that interfere with activation of replication stress response (37,38). To help navigate through error prone sequences in condensed chromatin, HC replication is facilitated by factors that help overcome these limitations including translesion DNA synthesis polymerase zeta (39) and topological sensor TRF2 (40).

A commonly employed replication stress response pathway is replication fork reversal (41). In this process, the newly synthesized DNA strands are extruded and reannealed to generate a four-way structure resembling a Holliday junction. Reversed forks are generated by DNA translocases SMARCAL1, ZRANB3, HLTF and recombinase RAD51 (42). While fork reversal allows DNA lesions to be repaired or bypassed, the regressed fork serves as a substrate for DSB repair proteins which can be degraded by nucleases if left unprotected. HR pathway protein BRCA2 recruits RAD51 to stalled replication forks and stabilizes the regressed arm by forming stable RAD51 nucleoprotein filaments (43,44). Inadequate fork protection results in nucleolytic degradation of nascent DNA that is broadly dependent on MRE11 or DNA2 nucleases. In cells devoid of BRCA2 function, reversed forks are subjected to degradation by MRE11 and EXO1 (45). Furthermore, inactivating DNA translocases rescues nascent strand degradation in BRCA2-deficient cells indicating that reversed forks act as substrates for nuclease-mediated DNA digestion (46,47). Deregulation in stalled fork protection causes genome instability and is a predicted determinant of cellular fitness, chemosensitivity and inflammation. Fork protection is regulated by several factors and pathways that also function in DSB repair (48,49), but the influence of chromatin state on stalled fork protection remains unexplored.

Errors that arise during DNA replication and escape proofreading are resolved by DNA mismatch repair (MMR) (50,51). MMR, which is highly conserved from bacteria to humans, is critical to replication fidelity since MMR deficiency results in mutator phenotype commonly associated with microsatellite instability and increased predisposition to multiple cancers (52,53). Mechanistically, MMR is an excision-gap filling synthesis pathway that recognizes base-base mismatches and small insertion/deletion loops (IDLs) (50). Mismatch-binding factors in human cells include MSH2, MSH3 and MSH6 that participate in MMR by forming heterodimer complexes MutSα (MSH2/6 dimer) and MutSβ (MSH2/3 heterodimer). Mismatch-bound MutS complexes recruit MLH1-containing MutL heterodimers that aid in DNA incision followed by degradation of the nicked strand to excise the mismatch. Discontinuities in the lagging strand (54,55) and asymmetric association of PCNA with replication forks (56) serve as strand discrimination signals to ensure excision of lesion occurs in the nascent strand thereby restricting the use of parental DNA as template. Apart from correcting replicative errors, MMR also affects fidelity of mitotic recombination by suppressing heteroduplex formation during homologous recombination (57,58).

Herein, we investigated whether replication fork protection is differentially regulated in late S-phase which coincides with duplication of heterochromatic DNA. Using synchronized human telomerase-immortalized retinal pigment epithelial (hTERT-RPE-1) cells coupled with DNA fiber analyses, we find replication forks exhibit similar replication elongation rates in early and late S-phase. While stalled fork protection remains intact in early S-phase, stalled forks in late S-phase are susceptible to nascent DNA digestion. Replication fork remodeling is a prerequisite for nascent strand degradation since fork protection is restored by inactivating either fork reversal enzymes or resection nucleases. Acute compaction of chromatin triggers digestion of nascent DNA with similar genetic dependencies indicating that fork protection is inherently curtailed in condensed chromatin regions. Mechanistically, chromatin compaction hinders RAD51 association with stalled forks indicating that RAD51 accessibility is a critical determinant of fork protection. Furthermore, MMR pathway dictates nascent strand susceptibility to DNA digestion at stalled forks during late replication. Independent of chromatin state, MMR inactivation alleviates nascent DNA digestion in BRCA2-deficient cells revealing an antagonistic function of MMR in fork protection. These studies elucidate regulatory mechanisms of protecting stalled forks in EC and HC and identify MMR as a mediator of nascent strand degradation.

## Materials and methods

### Cell culture and cell synchronization

hTERT-RPE-1 cells were cultured in DMEM/F12 media (Gibco 11320033), HCT116 cells were cultured in McCoy 5A media (Gibco 16600108) and U2OS cells were cultured in DMEM media (Gibco 11965092), each supplemented with 7.5% FBS (RD Systems S11150) and 1% penicillin/streptomycin (Gibco 15140-122) at 37°C with 5% CO_2_. For cell synchronization assays, RPE-1 and HCT116 cells were grown to 80–90% confluency before being placed in serum-free media with 1% penicillin/streptomycin for 24 h. To release cells from synchronization, cells were collected using trypsin + 0.05% EDTA (Gibco 25300120) and plated in serum-containing media. Synchronized RPE-1 cells were collected at 18 and 32 h post-serum addition to analyze replication in early and late S-phase respectively. Synchronized HCT116 cells were collected at 8 and 16 h post-serum addition to analyze replication in early and late S-phase respectively.

### DNA fiber assay

2.5 × 10^5^ cells were plated in a 6-well dish the day before collection. Conditioned media and 1× HBSS were equilibrated to 37°C with 5% CO_2_ overnight. On the day of collection, cells were pulse-labeled for 30 min with 20μM CldU, washed 2 times with 1× HBSS and pulse-labeled with 100 μM IdU for 30 min. For fork elongation assays, cells were collected and resuspended for DNA fiber analyses. For nascent strand degradation assays, cells were washed 2 times with 1× HBSS and treated with either hydroxyurea (HU) or aphidicolin (APH) for the indicated times. For fork recovery assays, cells were first pulse-labeled with 20μM CldU for 30 min, washed 2 times with 1× HBSS and treated with HU alone or HU and ATR inhibitor (ATRi) for 4 h. After treatment, cells were washed 2 times with 1× HBSS and pulse-labeled with 100 μM IdU for 30 min before collection. After collection, cells were resuspended in ice-cold 1× DPBS. To compensate for the lower number of replicating cells in the synchronized populations, cells harvested at the 18-h and 32-h time points were resuspended at roughly 5-fold higher concentration compared to asynchronous cells.

To spread DNA fibers, 2–4 μl of cell suspension was added to slides. Slides were incubated at room-temperature (RT) for 6 min. To lyse cells, 10 μl of spreading buffer (50 mM EDTA, 200 mM Tris–HCl pH 7.4, 0/5% SDS) was added, pipetting up and down 5–10 times to ensure proper lysis and incubated at RT for 6 min. Slides were then tilted allowing DNA to migrate slowly to the end of the slide. Slides were allowed to dry for 30 min before being fixed in 3:1 methanol:glacial acetic acid in coplin jars for 2 min. Slides were then dried at RT for 10 min and stored at −20°C overnight. Once removed from −20°C, DNA fibers were rehydrated in 1× PBS for 5 min. DNA was then denatured in 2.5 N HCl for 37 min at RT. Slides were rinsed 3 times with 1× PBS. Slides were incubated in PBST + Goat Serum (1× PBS + 0.1% TX-100 + 10% goat serum) for 1 h at RT. Slides were incubated with primary antibodies for 2 h at RT. Slides were washed 3 times with 1× PBS before incubation with secondary antibodies for 1 h at RT. Slides were rinsed 3 times with 1× PBS and mounted with coverslips using ProLong Gold Antifade Mountant. Slides were imaged using a 60X oil objective on the Keyence BZ-X810 All-in-One Fluorescence Microscope. Fibers were measured using ImageJ software.

### DNA combing assay

All protocols and reagents were utilized from Genomic Vision. Briefly, 2.5 × 10^5^ cells were plated the day before collection. On the day of collection, cells were pulse-labeled for 30 min with 20 μM CldU. Cells were washed 2 times with 1× HBSS and pulse-labeled with 100 μM IdU for 30 min. Cells were then collected and resuspended in resuspension buffer (Genomic Vision MCS-001) before being embedded into agarose plugs. Plugs were incubated with protease overnight at 50°C. Next day, plugs were washed 3 times in wash buffer before incubation in agarose overnight at 42°C. DNA was transferred into reservoirs (Genomic Vision RES-001) the following day and combed onto CombiCoverslips (COV-002-RUO) using the FiberComb® molecular combing system. Coverslips were then dried at 60°C for 2 h and stored at −20°C.

Coverslips were dehydrated in ethanol (70%, 90% and 100%, successively) for 3 min each before being dried at RT. DNA was denatured in 0.5 M NaOH/1 M NaCl for 8 min at RT. Coverslips were washed 3 times in 1× PBS before being dehydrated (stated above). Coverslips were first incubated in primary antibody solution in Block Aid for 1 h at 37C, then washed 3 times in 1× PBS + 0.05% Tween20 for 3 min each and incubated in secondary antibody solution in Block Aid for 45 min at 37°C before being washed 3 times in 1× PBS + 0.05% Tween20 for 3 min each. To stain total DNA, coverslips were incubated for 1 h at 37°C in 2:25 Mouse anti-ssDNA. After being washed 3 times in 1× PBS + 0.05% Tween20, ssDNA was counterstained with 2:25 Goat anti-Mouse BV480. Slides were imaged using the Genomic Vision FiberVision®S Automated Scanner equipped with a 40× objective lens and analyzed using FiberStudio® Version 3.3.5.

### Click reactions and SIRF assay

Cells seeded on coverslips were pulse-labeled for 1 h with 10 μM 5-ethynyl-2′-deoxyuridine (EdU) for immunofluorescence assays or 125 μM EdU for *in situ* analysis of protein interactions at DNA replication forks (SIRF) assays. Coverslips were rinsed with 1× DPBS (Gibco 14190250) and permeabilized with Triton X-100 permeabilization buffer (20 mM HEPES, 50 mM NaCl, 3 mM MgCl_2_, 300 mM sucrose, 0.5% Triton X-100) for 10 min at 4C or on ice. Cells were rinsed 3 times with 1× DPBS and fixed using 3% PFA/2% sucrose for 10 min at RT and stored at 4°C. Before antibody staining, coverslips were permeabilized a second time using Triton X-100 permeabilization buffer and blocked in 5% bovine serum albumin (BSA) for 15 min each at RT. The EdU-click reaction was then performed (875 μl 1×-PBS, 5 μl Alexa Fluor™ 488 or Alexa Fluor™ 594, 100 μl 20 mg/ml sodium ascorbate and 20 μl 100 mM copper sulfate) at RT for 30 min in the dark. For SIRF assays, Biotin-Azide was used instead of AF488/594 in click reactions. Coverslips were then incubated with primary antibodies diluted in 1× antibody dilution buffer overnight at 4°C containing the following primary antibodies: Rabbit anti-RAD51 at 1:400, Mouse anti-Biotin at 1:200 and Rabbit anti-MRE11 at 1:200. In Situ PLA was then performed utilizing Duolink PLA Red (Sigma Aldrich DUO92101). Coverslips were mounted onto slides using Prolong Gold Antifade Mounting Media with DAPI (Invitrogen P36931). Samples were imaged using a 40× objective on the Keyence BZ-X810 All-in-One Fluorescence Microscope. Hybrid Cell Count module on the Keyence BZ-X analyzer software was used to quantify PLA foci.

### Immunofluorescence MNase assays

Cells were seeded at 3 × 10^5^ on glass coverslips the day before treatment. Cells were pulse-labeled with 10 μM EdU for 1 h followed by permeabilization in 1 ml Triton X-100 permeabilization buffer (20 mM HEPES, 50 mM NaCl, 3 mM MgCl_2_, 300 mM sucrose, 0.5% Triton X-100) for 10 min at 4°C or on ice. Cells were rinsed 3 times with 1× PBS and treated with 100 units MNase in 2 ml MNase Buffer (0.32 M sucrose, 50 mM Tris–Cl pH 7.5, 4 mM MgCl_2_, 1 mM CaCl_2_, 0.1 mM PMSF) for 30 min at RT. Cells were rinsed 3 times with 1× PBS and incubated for 10 min on ice with Triton X-100 permeabilization buffer supplemented with 5 mM EGTA to inactivate the MNase enzyme. After rinsing cells 3 times with 1× PBS, cells were fixed in 3% PFA/2% sucrose for 10 min at RT. Cells are permeabilized with Triton X-100 permeabilization buffer for 15 min on ice. After 3 washes with 1× PBS, cells were blocked in 5% BSA/PBS for 15 min at RT. The EdU-click reaction was performed, and coverslips mounted on slides using Prolong Gold Antifade Mounting Media with DAPI.

### Protein extraction and immunoblotting

Cells were incubated on ice for 30 min in 4–5 times the volume of RIPA lysis buffer (50 mM Tris–Cl pH = 7.4, 1% NP-40, 150 mM NaCl, 0.1% SDS, 1 mM DTT, 0.5% sodium deoxycholate, 1 mM aprotinin, 1 mM leupeptin, 1 mM PMSF, 20 mM beta glycerol phosphate, 1 mM MgCl_2_ and 1 mM sodium orthovanadate) supplemented with 1 μg/ml Pierce Universal Nuclease (ThermoFisher 88701). After centrifugation at 13 200 rpm for 15 min at 4°C, the supernatant was collected and protein concentration was quantified by spectrophotometry (Biotek Epoch Spectrophotometer). 30–100 μg of whole cell lysates were run on SDS-PAGE gels and proteins were transferred to nitrocellulose membranes overnight. Membranes were blocked using 5% milk in TBST for 1 h, followed by probing with primary and secondary antibodies in 1% milk in TBST. Membranes were imaged using near-infrared (NIR) fluorescent imager (Licor Odyssey DLx) equipped with Image Studio.

### siRNA and plasmid transfections

RPE-1 cells were transfected with siRNA using RNAi MAX (Invitrogen 13778075) and 40 pmol siRNA in Opti-MEM Reduced Serum Media (Gibco 31985070) for 48–96 h. For plasmid transfections, RPE-1 cells were transfected using Lipofectamine 2000 (Invitrogen 11668027) in Opti-MEM Reduced Serum Media for 48–72 h using the following constructs: CMV-hRAD51 (Addgene Plasmid #125570), CMV-hRAD51K133R (Addgene Plasmid #125571), CMV-MSH2 Myc-DDK-tagged (Origene RC205848) and CMV-MLH1 Myc-DDK-tagged (Origene RC201607).

### Antibodies

For immunofluorescence studies, the following antibodies were used: Rat monoclonal anti-BrdU 1:100 (Abcam ab6326); Mouse anti-BrdU BD Biosciences 1:10 (347580); Alexa Fluor™ 488 (Invitrogen A10266) or Alexa Fluor™ 594 (Invitrogen A10270); (Goat anti-Rat Cy5 2:25 (Abcam ab6565), Goat anti-mouse Cy3 2:25 (Abcam ab97035); Rabbit anti-phospho histone H3 Ser 10 (Cell Signaling Technology 9701S) 1:200, Rabbit anti-RAD51 (Abcam ab133534); Rabbit anti-MRE11 (Cell Signaling Technology 4895S), Mouse anti-Biotin (Jackson Immuno 200-002-211); Duolink PLA Red (Sigma Aldrich DUO92101); Mouse anti-ssDNA (DSHB AUTOANTI-SSDNA-S); Goat anti-Mouse BV480 (Jackson 115-685-166); Invitrogen Goat anti-mouse IgG AF488 1:175 (A-11001); Invitrogen Goat anti-Rat IgG AF594 1:350 (A-11007). For immunoblotting, the following antibodies were used: Anti-BRCA2 (Ab-1) Mouse mAb (2B) (Millipore OP95-100UG) 1:500; Mouse anti-MSH2 (Santa Cruz sc-376384) 1:1000; Rabbit anti-MLH1 (Abcam ab92312) 1:1000; Rabbit anti-DCAF14 (Novus Biologicals NBP2-33883) 1:500; Rabbit anti-KU70 (Abcam ab92450) 1:7000; Mouse anti-cyclin A (B-8) (Santa Cruz sc-271682) 1:1000; Rabbit anti-cyclin B1 (Cell Signaling Technologies 4138S) 1:1000; Mouse anti-cyclin E1 (Cell Signaling Tech 4129T) 1:1000; Mouse anti-PCNA (Santa Cruz sc-56) 1:1000; Mouse anti-SMARCAL1 (Santa Cruz sc-376377) 1:1000; Rabbit anti-HLTF (Abcam ab13042) 1:1000; Rabbit anti-ZRANB3 (Bethyl A303-033A) 1:1000; Rabbit anti-RAD51 (Abcam ab133534) 1:1000; Rabbit anti-MRE11 (Cell Signaling Technology 4895S) 1:250; Rabbit anti-DNA2 (Invitrogen PA5-77943) 1:250; Rabbit anti-EXO1 (Bethyl A302-640A) 1:2000; Rabbit anti-Histone H3 (Abcam ab1791) 1:10 000; IR Dye 680 LT Donkey anti-mouse (LI-COR 925-68022); IR Dye 680 LT Donkey anti-rabbit (LI-COR 925-68023); IR Dye 800 CW Donkey anti-mouse (LI-COR 925-32212); IR Dye 800 CW Donkey anti-rabbit (LI-COR 925-32213).

### siRNAs

Non-targeting (NT) (Dharmacon D-001810-01-20); SMARCAL1 (J-013058-06-0005); HLTF (Dharmacon L-006448-00-0005); ZRANB3 (Dharmacon D-010025-03-0005); MRE11 (Dharmacon J-009271-08-0002); DNA2 (Dharmacon D-026431-03-0002); EXO1 (Dharmacon L-013120-00-0005); MSH2 (Dharmacon L-003909-00-0005); MLH1 (Dharmacon J-003906-09, J-003906-10, J-003906-11, J-003906-12, pooled); RAD51 (Dharmacon J-003530-11), BRCA2 (Qiagen SI02653434) and DCAF14 (Dharmacon J-019291-06).

### Compounds

Rad51 inhibitor B02 (Sigma-Aldrich SML0364); Mre11 inhibitor Mirin (Selleck Chemicals S8096); DNA2 inhibitor C5 (MedChem Express 35973-25-2); PARP inhibitor Olaparib (Selleck Chemicals S1060); Hydroxyurea (HU) (Sigma Aldrich 127-07-1); Aphidicolin (APH) (Sigma Aldrich A0781-5MG); Camptothecin (CPT) (Sigma Aldrich 7689-03-4); Trichostatin A (TSA) (Selleck Chemicals S1045); ATRi VE-821 (Selleck Chemicals S8007); Sucrose (Fisher Chemical S5-3); CldU (5-chloro-2′-deoxyuridine, Sigma-Aldrich C6891); EdU (5-ethynyl-2′-deoxyuridine, Sigma-Aldrich 61135–33-9); IdU (5-iodo-2′-deoxyuridine, Sigma-Aldrich I7125).

### Data analysis

All statistical analyses were performed using GraphPad Prism 10. Details of the statistical tests are included in the individual figure legends. For all statistical measures, significance values were derived using *P* value = 0.05 as cutoff. Representative experiments are shown, unless otherwise indicated, and all experiments were performed at least twice.

## Results

### Enrichment of replication forks from early and late S-phase

**Figure 1.**
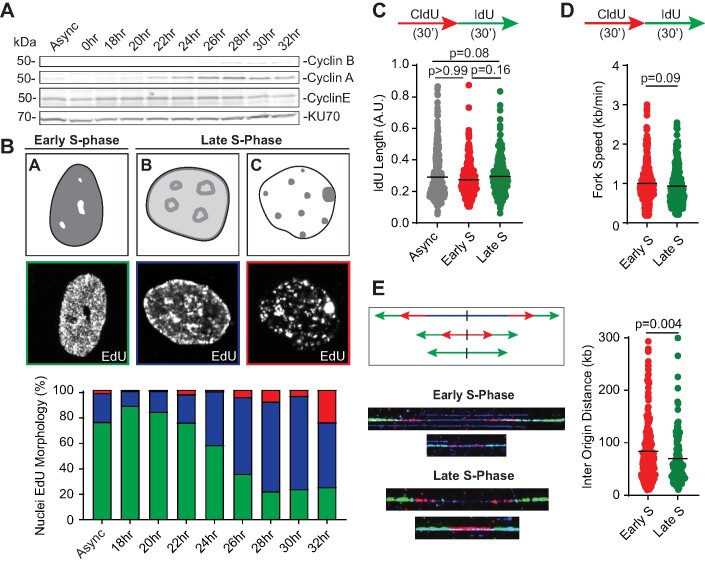
Single molecule analyses of DNA replication in early and late S-phase using synchronized RPE-1 cells. (**A**) Whole cell lysates were extracted from RPE-1 cells at the indicated time points during S-phase after release from serum starvation and analyzed by immunoblotting. KU70 serves as a loading control. (**B**) Top panel: Representative cartoons and confocal images for EdU patterns in subtypes A, B and C are depicted. Subtype A represents early replication while subtypes B and C represent late replication. Gray shades denote different EdU intensities. Bottom panel: Bar graph represents percent (%) nuclei for subtypes A (green), B (blue) and C (red) at the indicated timepoints in synchronized RPE-1 cells. (**C**) DNA fiber analysis was performed using asynchronous RPE-1 cells (Async), and synchronized RPE-1 cells collected after release from serum starvation at 18 h for early replication and 32 h for late replication. IdU lengths are plotted (A.U.= arbitrary units). (**D, E**) DNA combing analysis was performed using synchronized RPE-1 cells collected at 18 h (early S) and 32 h (late S) after release from serum starvation. In panel D, dual labeled replication tracts were analyzed, and fork speeds (kb/min) are plotted. In panel E, bidirectional replication tracts were analyzed and inter-origin distances (kb) are plotted. Cartoons and representative images for origins are depicted. p-values were derived using Kruskal–Wallis test with Dunn's multiple comparisons in panel (C) and Mann–Whitney test in panels (D) and (E). Black horizontal lines represent mean.

DNA replication is coordinated both spatially and temporally, with euchromatin (EC) regions replicating in early S-phase while heterochromatin (HC) replication predominantly occurring in mid to late S-phase (59,60). To explore replication fork dynamics during early and late replication, we utilized serum starvation to obtain synchronized RPE-1 cells. RPE-1 cells were selected since they represent near diploid, karyotypically stable cell lines without any transformed phenotypes (61). Cells were arrested in G0 phase by serum deprivation for 24 h and released into serum-rich media to stimulate cell cycle entry. In comparison to the asynchronous population, cells collected post 24 h of serum-starvation have low levels of cyclin E and cyclin A (Figure 1A, compare Async to 0 hour) indicating that replication is largely absent after serum deprivation. Following release into serum-rich media, cells were collected every 2 h beginning at 18 h until 32 h post-serum addition. To determine which timepoints best correspond to early and late S-phase, synchronized cells were first analyzed for cyclin levels by immunoblotting. Replicating population is detectable at the 18-hour time point as indicated by presence of S-phase cyclin A (Figure 1A). Conversely, mitotic cyclin B appears as early as 26 h indicating a population shift to late S-G2 phase. Next, to determine sub-populations of S-phase in synchronized cells, we performed immunofluorescence analysis to visualize replication patterns using EdU-based click chemistry. Chromatin folding generates two distinct nuclear compartments A and B. Compartment A correlates with open EC that replicates early in S-phase while compartment B represents late-replicating, compact HC (9). EdU patterns within asynchronous population are distinguishable as subtypes A, B and C, thus allowing us to identify replication of chromatin compartments within S-phase. Early replication appears more diffused (Figure 1B, subtype ‘A’), while heterochromatic regions in mid and late S-phase exhibit replication patterns predominately at nuclear periphery, nucleolar periphery and as chromocenter/pericentromeric bodies (Figure 1B, subtypes ‘B’ and ‘C’). Using this labeling strategy with the synchronized populations, we find that the majority of EdU-positive cells at the 18-hour time point (∼90% cells, Figure 1B, bottom panel) display pan-nuclear subtype ‘A’ pattern. By following cellular progression every 2 h from early S-phase to late S-phase, subtype ‘A’ starts to decline and by 32 h post-serum addition, S-phase nuclei have predominately HC associated subtypes ‘B’ and ‘C’. Thus, by 32 h post serum addition, replicating cells are predominately in late S-phase undergoing HC replication while a large proportion of cells have transitioned into G2/M, consistent with a drop in Cyclin A levels at roughly 30 h (Figure 1A). EdU + nuclei in asynchronous, early and late S-phase populations lack detectable phospho-histone H3 (Ser 10) counterstain indicating that replicating cells are not in mitosis (Supplementary Figure S1A). The enrichment of early and late replicating cells at 18 and 32 h respectively was also corroborated by flow cytometry analysis (Supplementary Figure S1B). For all experiments outlined below, we visualized EdU patterns to measure S-phase distributions and changes in replication compartments in conditions of either genetic depletions or drug treatments. Respective tabular analysis showing S-phase distributions for all experiments is included in Table S1.

### Analyses of replication dynamics between early and late S-phase

To investigate replication fork dynamics between early and late S-phase, we analyzed single replicating molecules of DNA using both DNA spreading and DNA combing techniques. Synchronized populations at 18 and 32 h were sequentially pulsed with thymidine analogs CldU and IdU for 30 min each. Analysis of dual labeled replication tracts demonstrate that replication forks in early and late replication elongate at largely similar rates (Figure 1C and D). Using DNA combing methodology, we then assessed the density of origins in synchronized cells from early and late replicating populations. Measurement of inter origin distances reveals that late replicating cells are marginally denser in origins compared to early replicating cells (Figure 1E). Thus, replication fork populations analyzed at 18 and 32 h are primarily denotive of early and late replicating regions respectively. While fork elongation is minimally perturbed in HC, it is possible that replication is altered in a sequence- or loci-dependent context (33).

### Replication forks in late S-phase are susceptible to nascent strand degradation

**Figure 2.**
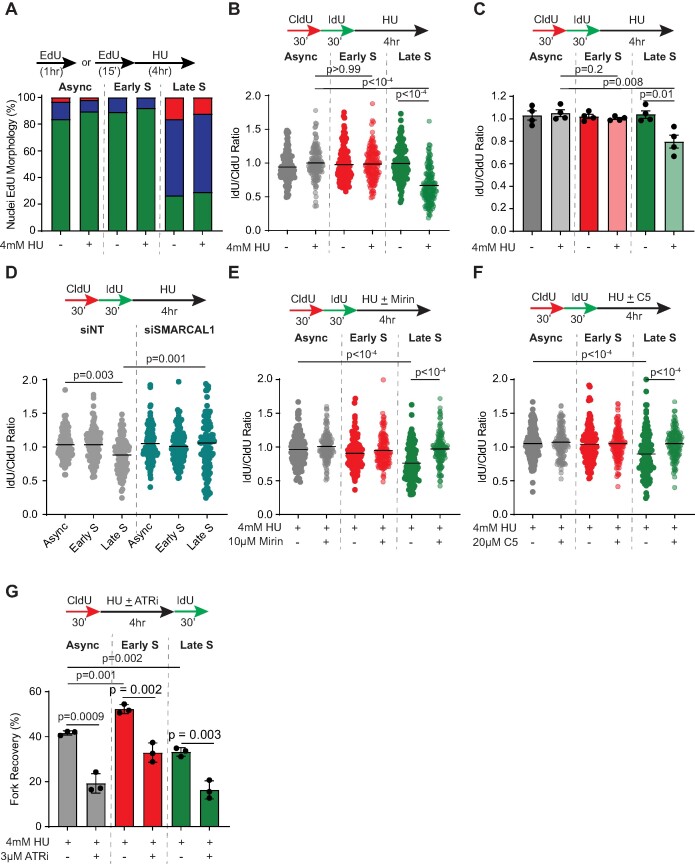
Stalled forks in late replication are susceptible to nascent strand degradation. (**A**) Bar graph represents percent (%) nuclei distribution for subtypes A (green), B (blue) and C (red) in either asynchronous (Async) RPE-1 cells, early S-phase (Early S) or late S-phase (Late S) RPE-1 cells harvested post serum starvation at the indicated time points. Cells were left untreated or treated with HU for 4 h. **(B, C)** Fork degradation assays were performed in Async, Early S or Late S RPE-1 cells as indicated. Representative IdU/CldU ratios from 1 biological replicate is depicted in panel B. IdU/CldU ratios from 4 biological replicates are depicted using mean ± S.E.M in panel C. (**D–F**) Fork degradation assays were performed in Async, Early S or Late S RPE-1 cells as indicated. In panel D, cells were transfected with either non-targeting siRNA (siNT) or siRNAs targeting SMARCAL1 (siSMARCAL1). In panels E and F, cells were concomitantly treated with or without MRE11-inhibitor mirin (E) and DNA2-inhibitior C5 (F). Representative IdU/CldU ratios are plotted. **(G)** Fork recovery assays were performed in Async, Early S or Late S RPE-1 cells as indicated. Cells were treated either with HU alone or HU with ATR inhibitor. Replication tracts with both IdU and CldU labels were quantified as percentage of all fibers analyzed. Graph represents mean ± S.E.M. (*n* = 3). *P*-values were derived using Kruskal–Wallis test with Dunn's multiple comparisons in panels (B), (D), (E) and (F), and unpaired *t* tests in panels (C) and (G). Black horizontal lines represent mean.

Chromatin organization and nuclear positioning can influence DNA repair and resolution of replication stress (20). A common response to replication fork obstacles is the remodeling of forks to four-way junctions to generate reversed replication forks (62–64). Multiple stress-response factors act at remodeled forks to protect the regressed arm from nuclease-mediated digestion in a process termed replication fork protection. Whether replication fork protection is altered in late replication is unknown given that asynchronous RPE-1 cells represent forks in early replicating regions (Figure 1B, compare Async versus 18 h) thereby obscuring the consequences at stalled forks in late-replicating regions. Using synchronized populations, we performed nascent strand degradation assays to determine whether protection of newly synthesized DNA is differentially regulated between early S-phase and late S-phase. Early and late replicating populations obtained from synchronized RPE-1 cells (Figure 2A) were sequentially pulsed with CldU and IdU for 30 min each followed by exposure to high-dose (4 mM) HU for 4 h (Figure 2B and C). Asynchronous RPE-1 cells exhibit IdU/CldU ratio of ∼1 indicating stalled forks remain protected after HU exposure. While HU-exposed nascent DNA remains intact at early replicating forks, late replicating forks display nascent strand shortening as measured by reduced IdU/CldU ratios after HU treatment (Figure 2B). The reduced nascent DNA stability in late S-phase is observable across multiple biological repeats (Figure 2C) and is not due to major alterations in chromatin organization since EdU patterns in S-phase synchronized populations continue to persist even in presence of HU (Figure 2A). Additionally, nascent strand degradation assays at 20, 24 and 28 h post serum starvation reveal a gradual loss in stalled fork protection indicating a strong correlation between nascent strand degradation and forks derived from late replication (Supplementary Figure S2A). To rule out the possibility that these observations arise in conditions of HU exposure alone, we assessed susceptibility of stalled forks to nascent strand degradation in early and late replicating regions using high dose and low dose aphidicolin. For high dose conditions, we adopted 10 μM aphidicolin previously shown to trigger nascent DNA digestion (65). 0.4 μM aphidicolin was used as low dose, which characteristically triggers replication stress at fragile sites to induce chromosomal breakage (66). 10μM aphidicolin induces degradation of nascent DNA at all replication forks regardless of the timing in S-phase (Supplementary Figure S2B). While stalled fork protection in presence of 0.4μM aphidicolin is intact in early replicating cells, late replicating cells exhibit nascent strand degradation in response to low dose aphidicolin (Supplementary Figure S2B) further supporting the finding that stalled forks undergo nascent strand degradation in late replicating, HC regions.

Substrates for nascent strand degradation are reversed replication forks that are generated by translocases SMARCAL1, ZRANB3 and HLTF (64). Thus, we transiently depleted the fork reversal enzymes to establish whether remodeled replication forks are required for nascent DNA digestion in late replication. Removal of either SMARCAL1, HLTF or ZRANB3 is sufficient to restore replication fork protection (Figure 2D and Supplementary Figure S2C). Exposure to PARPi, previously shown to restart reversed replication forks (67), also restores fork protection thereby suggesting that reversed fork is a likely substrate for nascent strand degradation (Supplementary Figure S2D). To further confirm that the reduction in IdU/CldU ratio represents degradation of nascent DNA, we asked whether blocking the actions of DNA nucleases rescues nascent DNA stability. Resection nucleases MRE11 and DNA2 catalyze nascent DNA digestion when forks are unprotected (49). MRE11 or DNA2 inactivation by siRNA-depletion restores nascent stability when late S-phase forks are exposed to 4mM HU (Supplementary Figure S2E). Fork protection is also restored upon transient treatment with MRE11 inhibitor mirin, DNA2 inhibitor C5 or co-treatment with both nuclease inhibitors (Figure 2E, F and Supplementary Figure S2F) indicating that the nuclease activity of both MRE11 and DNA2 is responsible for nascent DNA digestion. Interestingly, while inhibition of DNA damage response kinase ATR curtails recovery of all stalled replication forks, as expected (68), the ability of late S-phase, HU-stalled forks to resume DNA synthesis is compromised when compared to stalled forks in both asynchronous and early-replicating cells (Figure 2G). Restoring fork protection by mirin also elevates stalled fork recovery in late S-phase (Supplementary Figure S2G). We conclude that stalled forks in late S-phase undergo replication fork remodeling in a manner dependent on fork reversal enzymes but are innately susceptible to nuclease-mediated degradation resulting in fork restart deficiency.

### Chromatin compaction impedes RAD51-dependent replication fork protection

**Figure 3.**
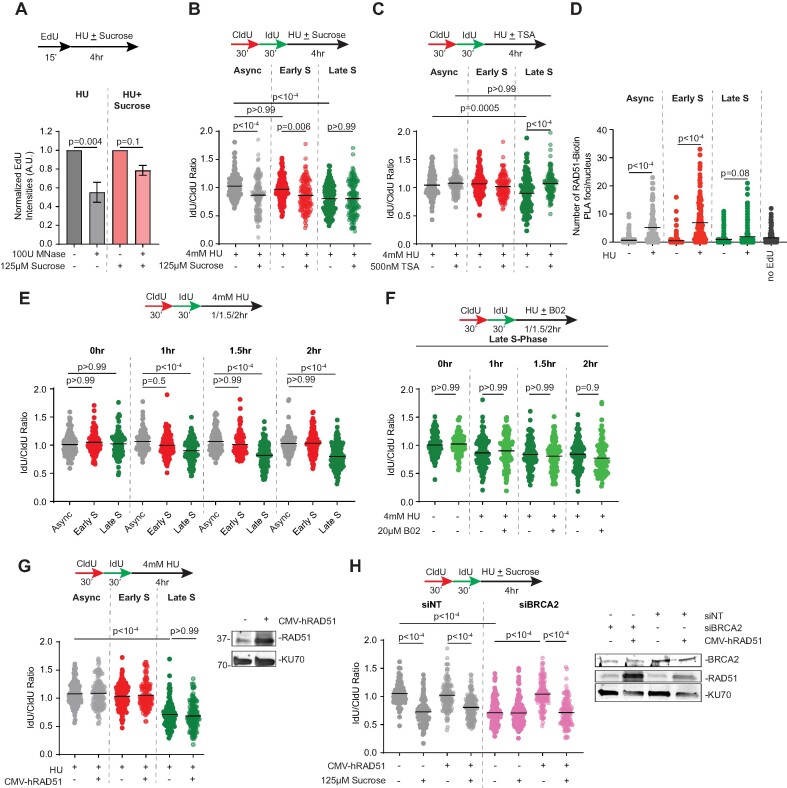
RAD51-dependent stalled fork protection is curtailed in condensed chromatin. (**A**) RPE-1 cells were pulse labeled with EdU followed by exposure to either HU alone or HU with sucrose for 4 h. Following permeabilization, cells were subjected to MNase digestion and reaction was quenched prior to fixation. Nuclear EdU intensities were quantified and normalized to samples that were not treated with MNase. Graphs represent normalized intensities with mean ± S.E.M (*n* = 3). (**B, C**) Fork degradation assays were performed in Async, Early S or Late S RPE-1 cells. Where indicated, cells were either treated with sucrose or trichostatin A (TSA). (**D**) Async, Early S or Late S RPE-1 cells were treated with or without HU and subjected to SIRF analysis using antibodies targeting RAD51 and Biotin. No EdU sample serves as a negative control. (**E**) Fork degradation assays were performed in Async, Early S or Late S RPE-1 cells in presence of HU for 1, 1.5 and 2 h. (**F**) Fork degradation assays were performed in late replicating RPE-1 cells in presence of either HU alone or HU and B02 for 1, 1.5 and 2 h. (**G**) Fork degradation assays were performed in Async, Early S or Late S RPE-1 cells transfected with or without RAD51 cDNA. Immunoblot depicts RAD51 overexpression with KU70 as loading control. (**H**) RPE-1 cells transfected with the indicated siRNAs were co-transfected with or without RAD51 cDNA and subjected to fork degradation assays. Where indicated, assays were performed in presence of sucrose. Representative immunoblot is shown with KU70 as a loading control. *P*-values were derived using unpaired *t* tests in panels (A) and (D), and Kruskal–Wallis test with Dunn's multiple comparisons in panels (B), (C), (E), (F), (G) and (H). Black horizontal lines represent mean.

The compartmentalization of HC domain due to compaction and phase separation alters availability of proteins and protein complexes (3). RAD51 is occluded from accessing resected DNA at double strand breaks in HC (15,16,20). In addition to homologous recombination (HR) repair of breaks, RAD51 also catalyzes nucleoprotein filament formation on regressed arms of reversed forks to protect from nuclease-dependent digestion (62). Since HC regions undergo duplication in late S-phase, we hypothesized that chromatin compaction creates a restrictive environment for stalled fork protection in a manner dependent on RAD51 association with perturbed forks. To test this, we first exposed asynchronous RPE-1 cells to hypertonic solution of sucrose to induce reversible molecular crowding (69). To confirm compaction of chromatin in response to sucrose, we designed an immunofluorescence-based quantitative imaging assay to detect changes in MNase sensitivity as a readout for chromatin accessibility in replicating cells. In this assay, EdU-labeled cells are incubated in presence or absence of sucrose, and following permeabilization, treated with MNase for 30 min. The MNase reaction is then quenched and fixed cells are analyzed to quantify nuclear EdU intensity. In cells treated with HU alone, EdU intensities reduce after MNase reaction indicating that chromatin is susceptible to MNase digestion (Figure 3A). In comparison, cells concomitantly treated with HU and sucrose show reduced sensitivity to MNase digestion indicating that sucrose causes condensation of DNA. We also find that cells retain the asynchronous EdU patterning after exposure to sucrose indicating that chromatin organization is minimally disrupted when chromatin is condensed transiently (Supplementary Figure S3A).

To evaluate the impact of chromatin compaction on fork protection, we treated asynchronous cells concurrently with 4mM HU and 125μM sucrose for 4 h. Remarkably, nascent DNA degradation is observed at both asynchronous and early S-phase forks treated with HU and sucrose (Figure 3B). This degradation is to the same extent to which late S-phase replication forks are digested in conditions of HU alone. Furthermore, exposing HU-stalled forks during late replication to sucrose does not exacerbate degradation, suggesting nascent DNA digestion is limited when chromatin is condensed (Figure 3B). If chromatin compaction is sufficient to cause nascent strand degradation, reversing the compacted state should restore replication fork protection at heterochromatic forks. To test this hypothesis, late S-phase synchronized cells were concomitantly treated with HU and trichostatin A (TSA), an HDAC inhibitor that rapidly induces histone acetylation to generate accessible chromatin and alleviates RAD51 accessibility at compact chromatin (20). Consistent with these observations, nascent strand degradation is abrogated at HU-stalled replication forks in presence of 500 nM TSA for 4 h (Figure 3C). Additionally, sucrose-induced digestion of nascent DNA is also blocked when either fork reversal enzymes are removed (Supplementary Figure S3B) or nucleases are blocked using small molecule inhibitors (Supplementary Figure S3C and S3D). Thus, similar to replication forks in late S-phase, excessive chromatin compaction creates a less permissive environment for stalled forks to protect newly synthesized DNA from digestion.

To determine whether chromatin compaction alters RAD51 association with sites of DNA synthesis, we measured RAD51 proximity to EdU-labelled DNA using *in situ* analysis of protein interactions at DNA replication forks (SIRF) assay (70). While asynchronous and early replication forks display increased RAD51 proximity to nascent DNA in presence of HU, RAD51 abundance is suppressed at late replication forks (Figure 3D). Similar results were also obtained in presence of replication stress reagent camptothecin (Supplementary Figure S3E). Furthermore, the HU-dependent increase in RAD51-Biotin proximity pairs is curtailed when sucrose is added (Supplementary Figure S3F). Conversely, acute TSA exposure increases RAD51 association with forks in late S-phase (Supplementary Figure S3G) indicating that heterochromatin regions in late S-phase curtail RAD51 localization to stalled forks. While RAD51 recruitment to HU-stalled forks in late replication is diminished, increased MRE11 recruitment to forks in response to HU occurs in both early and late S-phase cells (Supplementary Figure S3H). Given the limited association of RAD51 with stalled forks in late S-phase, we asked whether exposure to RAD51 inhibitor B02 enhances nascent strand degradation at stalled heterochromatic forks. To test this, we treated asynchronous, early S-phase and late S-phase synchronized populations with either HU alone or in presence of B02. Time course analysis in presence of HU shows a progressive decline in IdU/CldU ratios at stalled forks in late S-phase (Figure 3E). While HU-stalled forks undergo nascent DNA digestion when treated with B02, the extent of nascent DNA digestion in late S-phase is limited under these conditions (Supplementary Figure S3I) demonstrating a refractory nature of HC to RAD51-mediated replication fork protection. Consistent with this idea, the IdU/CldU ratios at stalled forks in late S-phase are not exacerbated with the addition of B02 across the time points tested (Figure 3F).

It is possible that limiting pools of RAD51 are available to successfully block nascent DNA from degradation at late replication forks. Thus, to test whether lack of fork protection at stalled forks is due to limited availability of RAD51 or restricted accessibility to RAD51, we ectopically expressed RAD51. Overexpression of RAD51 is not sufficient to alleviate nascent DNA digestion at stalled, late S-phase forks suggesting that chromatin compaction restrains RAD51 accessibility to replication forks in HC (Figure 3G). Fork protection continues to be impaired even when RAD51 K133R mutant, which is deficient in ATP hydrolysis but forms stable nucleoprotein filaments (71), is overexpressed (Supplementary Figure S3J). To further support the idea that RAD51 accessibility is curtailed in compact chromatin state, we overexpressed RAD51 in cells transiently transfected with siRNAs targeting either BRCA2 or DCAF14. In cells lacking BRCA2 or DCAF14 function, overexpressing RAD51 reverses nascent strand degradation (44,72). We then asked if transient chromatin compaction suppresses this rescue. In asynchronous BRCA2- and DCAF14-deficient cells, fork protection is restored when RAD51 is overexpressed (Figure 3H and Supplementary Figure S3K). However, adding sucrose during HU exposure evokes nascent DNA digestion in both BRCA2-silenced and DCAF14-silenced cells overexpressing RAD51. Overall, these results indicate that HC-like compacted state is unfavorable for RAD51-dependent replication fork protection.

### MMR components MLH1 and MSH2 antagonize BRCA2-mediated fork protection

**Figure 4.**
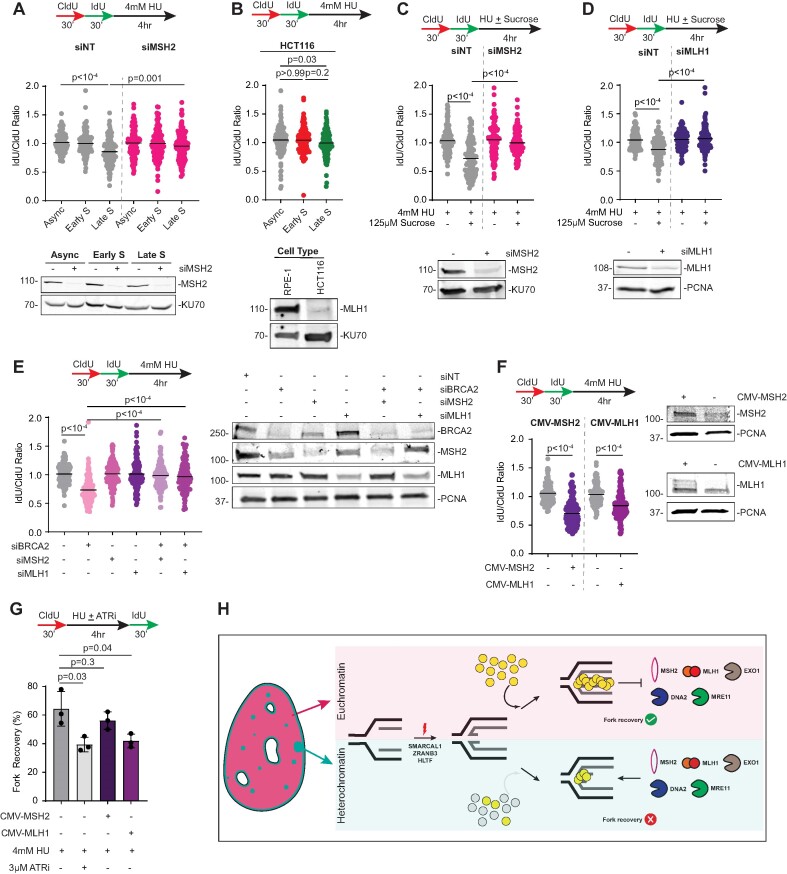
MMR pathway blocks BRCA2-mediated fork protection. (**A**) Fork degradation assays were performed in Async, Early S or Late S RPE-1 cells. Cells were transfected with either non-targeting siRNA (siNT) or siRNAs targeting MSH2 (siMSH2). Representative immunoblot is shown with KU70 as a loading control. (**B**) Fork degradation assays were performed in asynchronous HCT116 cells (Async), and synchronized HCT116 cells collected after release from serum starvation at 8 h for early replication and 16 h for late replication. Representative immunoblot is shown with KU70 as a loading control. (**C, D**) Fork degradation assays were performed in asynchronous RPE-1 cells transfected with the indicated siRNAs. Cells were treated with either HU alone or HU with sucrose as shown. Representative immunoblots are shown with either KU70 or PCNA as a loading control. (**E**) Fork degradation assays were performed in RPE-1 cells transfected with the indicated siRNAs. Representative immunoblot is shown with PCNA as a loading control. (**F**) Fork degradation assays were performed in RPE-1 cells transfected with either MSH2 cDNA or MLH1 cDNA. Representative immunoblots are shown with PCNA as a loading control. (**G**) Fork recovery assays were performed in asynchronous RPE-1 cells transfected with the indicated cDNAs. Replication tracts with both IdU and CldU labels were quantified as percentage of all fibers analyzed. Graph represents mean ± S.E.M (*n* = 3). *P*-values were derived using Kruskal–Wallis test with Dunn's multiple comparisons in panels (A), (B), (C) (D), (E) and (F), and unpaired *t* tests in panels (G). Black horizontal lines represent mean. (**H**) Fork reversal enzymes SMARCAL1, ZRANB3 and HLTF trigger fork remodeling when forks encounter obstacles in early replicating euchromatin and late replicating heterochromatin. The relatively open nature of euchromatin allows RAD51 to access the forks and protect regressed arms from digestion by resection enzymes and MMR thereby enabling fork restart. In comparison, the compact nature of heterochromatin restricts RAD51 accessibility and attenuates fork protection. As a consequence, reversed forks are degraded resulting in replication fork arrest. EXO1-dependent digestion of nascent DNA is triggered by MMR. For simplicity, MLH1 and MSH2 are depicted as heterodimers.

In addition to the DNA compaction environment, we asked whether nascent DNA stability at stalled forks during late replication is controlled by replisome components. Constitutive HC is mainly composed of repetitive DNA sequences that have the propensity to generate repeat instability by MMR (73). MMR pathway also has anti-recombinogenic functions at homologous and homeologous chromosomes (57,58) and is important in regulating HC stability (74). Since MMR components travel with replication forks (75–77), we asked whether MMR contributes to the strand degradation phenotype at stalled forks in late S-phase. First, we transiently depleted MSH2, the mismatch sensing component that forms heterodimers with MSH3 and MSH6. Silencing MSH2 restored fork protection at late replicating forks (Figure 4A). Next, to assess whether MMR pathway regulates stalled fork protection, we utilized MMR-deficient colon cancer HCT116 cell lines that lack constitutive MLH1 expression (Figure 4B) (78). Serum starved HCT116 cells were stimulated for re-entry into cell cycle and harvested at 8 and 16 h to study early and late replicating fork stalling events. EdU pattern analyses in HCT116 cells indicate that 8 and 16 h represent EC and HC replication patterns (Supplementary Figure S4A). Unlike HU-treated RPE-1 cells, stalled forks in late S-phase in HCT116 cells exhibit largely intact nascent strands thereby demonstrating a function of MMR pathway in antagonizing stalled fork protection in late S-phase (Figure 4B). Additionally, sucrose mediated digestion of nascent DNA is relieved when either MLH1 or MSH2 is depleted in asynchronous RPE-1 cells (Figure 4C and D). These data indicate that MMR facilitates nascent strand degradation in late replication.

Given the anti-recombinogenic functions of MMR in DSB repair, we asked whether MMR factors influence BRCA2-RAD51 dependent fork protection. We postulated that MMR-driven nascent strand degradation is not exclusive at forks in late S-phase but instead also manifests when RAD51-mediated filament formation is disrupted at stalled forks, independent of chromatin environment. To test this question, we co-depleted MSH2 or MLH1 in BRCA2-silenced, asynchronous RPE-1 cells. Transient depletion of BRCA2 induces fork degradation (Figure 4E) and reduces RAD51 presence at forks in cells treated with HU (Supplementary Figure S4B). In comparison, silencing either MSH2 or MLH1 restores fork protection in BRCA2-deficient cells (Figure 4E) without restoring RAD51 levels at forks to control levels (Supplementary Figure S4B). The rescue in nascent DNA digestion by MSH2 or MLH1 depletion is not due to alterations in RAD51 levels (Supplementary Figure S4C) and is also observable in U2OS cells (Supplementary Figure S4D). These results suggest that BRCA2-mediated fork protection is suppressed by MMR. MMR factors MSH2 and MLH1 are equally abundant at replication forks in early and late S-phase (77). Thus, we asked whether overexpression of MMR proteins triggers nascent strand degradation. We find that upregulating MSH2 or MLH1 is sufficient to abrogate fork protection in asynchronous RPE-1 cells (Figure 4F) and U2OS cells (Supplementary Figure S4E). MMR-mediated excision repair occurs in an EXO1-dependent or EXO1-independent manner (79). Nascent strand degradation assays in MLH1-overexpression conditions indicate that fork degradation is driven by EXO1 but is MRE11-independent (Supplementary Figure S4F). Furthermore, EXO1 depletion restores fork protection in late replication suggesting that MMR pathway mediates EXO1-dependent nascent strand degradation (Supplementary Figure S4G). Since late replication forks exhibit defective fork recovery, we tested whether MSH2 or MLH1 overexpression also compromises DNA synthesis post HU recovery. While stalled fork recovery remains unaffected in MSH2-overexpressing cells, fork restart is restricted in cells with upregulated MLH1 expression (Figure 4G). Overall, we conclude that MMR antagonizes BRCA2-dependent protection of nascent DNA.

## Discussion

Herein, we report that replication forks in late S-phase are defective in stalled fork protection. Since heterochromatin regions undergo duplication during late S-phase and represent dense regions of chromatin, the results suggest that heterochromatic forks are susceptible to nascent strand degradation in response to fork stalling events. Our study also shows that the degree of chromatin compaction impacts RAD51 accessibility to replication forks thus highlighting a critical link between chromatin environment and replication fork stability. Additionally, when BRCA2-mediated fork protection is compromised, shortening of nascent strands is driven by MMR indicative of BRCA2-RAD51 function in antagonizing MMR function to promote replication fork stability (Figure 4H).

Previous analyses of replication speed across S-phase have provided differing conclusions. Using DNA combing assays in synchronized RPE-1 cells to compare fork elongation rates in early-replicating and late-replicating regions, we find that fork speed is mostly unchanged throughout S-phase. These results are consistent with studies utilizing genome-wide replication timing and origin mapping data in combination with DNA combing data across multiple cell types (29). To capture forks from early and late replicating cells, we utilized serum starvation as cell synchronization strategy to minimize variable effects on replication timing or chromatin organization due to prolonged exposure to cell cycle arresting drugs such as thymidine and nocodazole. Furthermore, analyses of inter-origin distances from DNA combing experiments indicate that HC is origin dense relative to EC. Thus, large proportion of replication forks from synchronized populations are reflective of replicons that are enriched from distinct chromatin compartments.

Analyses of RAD51 association with nascent DNA reveals that stress dependent increase in RAD51-recruitment to sites of DNA synthesis is reduced in late replicating cells compared to early replicating cells. Furthermore, inducing chromatin compaction restricts RAD51 association with nascent DNA and blocks overexpressed RAD51 from rescuing nascent strand degradation in BRCA2- and DCAF14-deficient cells. Whether the stalled forks in late S-phase are relocalized in a manner similar to DSBs in HC (15,18,80) is not clear, but our results suggest that densely packed chromatin obstructs RAD51. Indeed, transient chromatin relaxation with TSA restores replication fork protection by increasing RAD51 abundance at late replicating forks and these observations are in agreement with the compacted state of HC shown to be refractory to RAD51 (15,16). Previous studies also demonstrate that RAD51 is almost always bound to BRCA2, irrespective of DNA damage conditions, and exist as large molecular weight BRCA2 clusters that are often multimeric (81,82) which likely limits diffusivity of RAD51 to HC. Heterochromatic DSBs in flies and mice move outside of heterochromatin to access RAD51 but the initial steps of HR repair, including resection, occur within the HC domains. Indeed, we find that both MRE11 and DNA2 contribute to nascent DNA digestion while EXO1-mediated digestion occurs through MMR. Additionally, fork reversal enzymes contribute to nascent strand degradation at HU-stalled heterochromatic forks and sucrose-treated forks indicating that the compact chromatin does not act as a barrier to fork remodeling enzymes. Presumably, the spatial uncoupling between early fork processing events and RAD51 recruitment to forks provides an opportunistic window for nucleases and MMR to degrade nascent DNA. Whether RAD51 actively contributes to replication fork remodeling in HC needs further investigation. Of note, while our analyses involve fork populations captured from late replication, it will be important to assess how fork protection is regulated in different HC environments since HC domains can be differentially repaired (19). Since our study utilizes sucrose to transiently induce chromatin compaction, additional studies are also necessary to investigate how compact state of heterochromatin directly impacts additional fork protection mechanisms.

Our findings indicate that digestion of nascent tracts in late replication is dependent on MMR. Since MMR factors travel with replication forks (75,76), differences in fork abundance of MMR components between early and late S-phase could impact replication fork protection. However, proteomic analyses indicate that MMR presence at replication forks remain unchanged across S-phase (77) which is expected given the role of MMR in resolving replication errors. Instead, we propose that the increased engagement of MMR at repetitive sequences in HC renders stalled forks to be vulnerable to nascent strand degradation (83). We speculate that MMR-mediated digestion of nascent DNA arises due to the detection of base-base mismatches or insertions/deletion loops (IDLs) (51,83) that arise on regressed arms of reversed replication forks. The regressed arm could represent pairing of homeologous DNA molecules, which act as preferred substrates for MMR rejection in bacteria (84).

Depleting MSH2 or MLH1 rescues nascent DNA digestion in BRCA2-deficient conditions indicating that MMR is a negative determinant of replication fork protection. During HR repair of DSBs, MMR promotes heteroduplex rejection to unwind the new duplex DNA after the strand invasion step (85,86). Repetitive rounds of rejection allow RAD51-bound 3′ overhang to pair with its identical homologous sequence to ensure error free repair of breaks. Thus, similar to negative regulation of HR, MMR factors may engage with the regressed arm of reversed forks and perturb RAD51-dependent protection of nascent DNA from nucleases. Fork reversal is considered to undergo several rounds of regression-restart events to ensure minimal recognition of regressed arms by DSB repair proteins (62,64) and such repetitions may help impede MMR capture of reversed forks. We also find that the nascent strand degradation in late-replication is dependent on MRE11, EXO1 and DNA2. The observed MRE11 and EXO1 dependency is consistent with their role in digesting nascent DNA at reversed forks in BRCA2-deficient cells (44). Furthermore, nascent DNA digestion in MLH1-overexpression conditions is mediated by EXO1 indicating that the EXO1-dependent MMR pathway (79) curtails fork protection. The role of DNA2, however, remains unclear.

Late-replicating HC is enriched for difficult-to-replicate, repetitive sequences such as telomeres (36) and certain CFSs (30,87,88) commonly undergo chromosomal breakage and trigger genomic rearrangements when replication is perturbed. We find that late-replicating forks are susceptible to nascent strand degradation not only in presence of HU, but low dose APH that induces chromosomal fragility (66). Intriguingly, recent studies demonstrate that several under-replicated regions in APH-treated cells coincide with mid or late S-phase and exhibit mitotic DNA synthesis (MiDAS) (89). Coupled with the observations in mice and flies that HC occludes RAD51 from breaks (14,18), defect in RAD51-mediated replication fork protection could enhance MiDAS in HC to complete DNA synthesis since RAD51-dependent fork protection inhibits RAD52-dependent break induced replication (90). Overall, our results may serve as a molecular basis for understanding how chromosome fragility arises in certain regions of DNA that are commonly associated with replication stress associated cancers.

In summary, we uncover intrinsic propensity of stalled forks in late S-phase to nascent strand degradation, identify MMR as a negative determinant of nascent DNA stability, and highlight functional significance of chromatin state on replication fork protection.

## Supplementary Material

gkae721_Supplemental_Files

## Data Availability

All data underlying this article will be shared by the corresponding author upon request.

## Notes

Present address: Erica Colicino-Murbach, Life Sciences Institute, University of Michigan, Ann Arbor, MI, USA.
