# Supplementary material for: Replication fork stalling in late S-phase elicits nascent strand degradation by DNA mismatch repair
Source: Nucleic Acids Res. 2024 Aug 24;52(18):10999–1013. doi: 10.1093/nar/gkae721 (PMC11472054; doi:10.1093/nar/gkae721)
Supplement: gkae721_Supplemental_Files [file gkae721_supplemental_files.zip › Supplementary_material.pdf]

Supplementary Figure S1

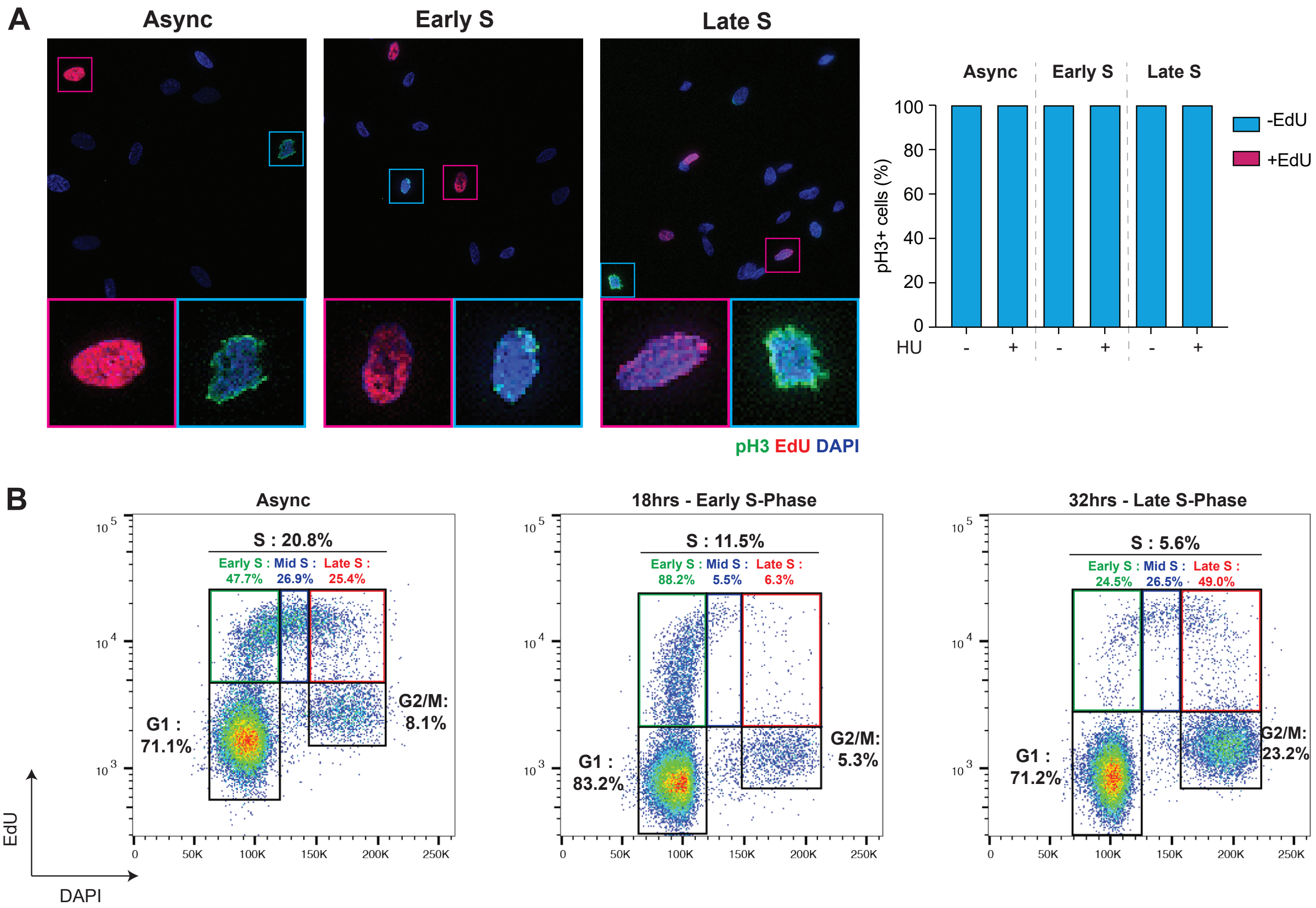

Supplementary Figure S2

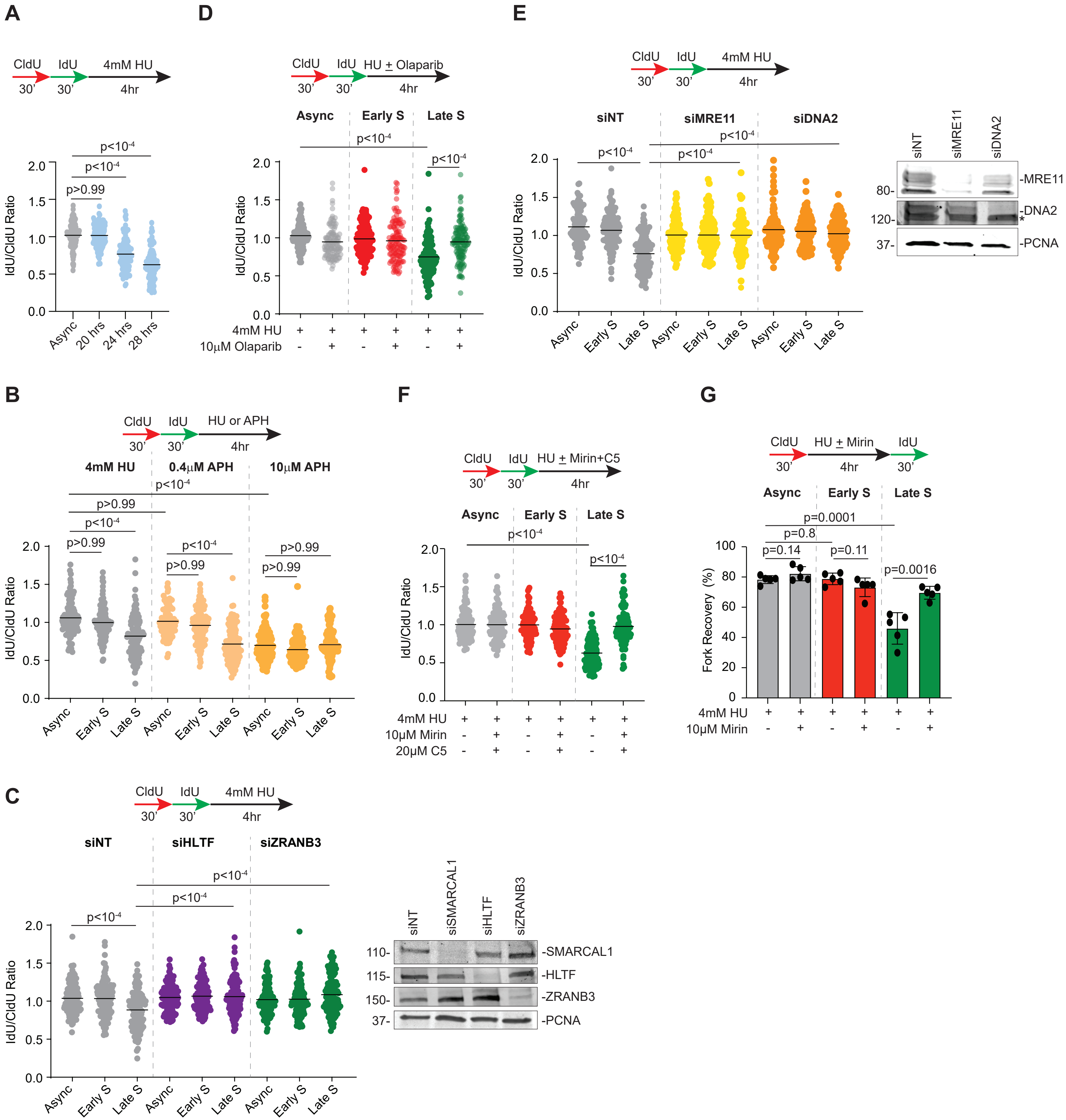

Supplementary Figure S3

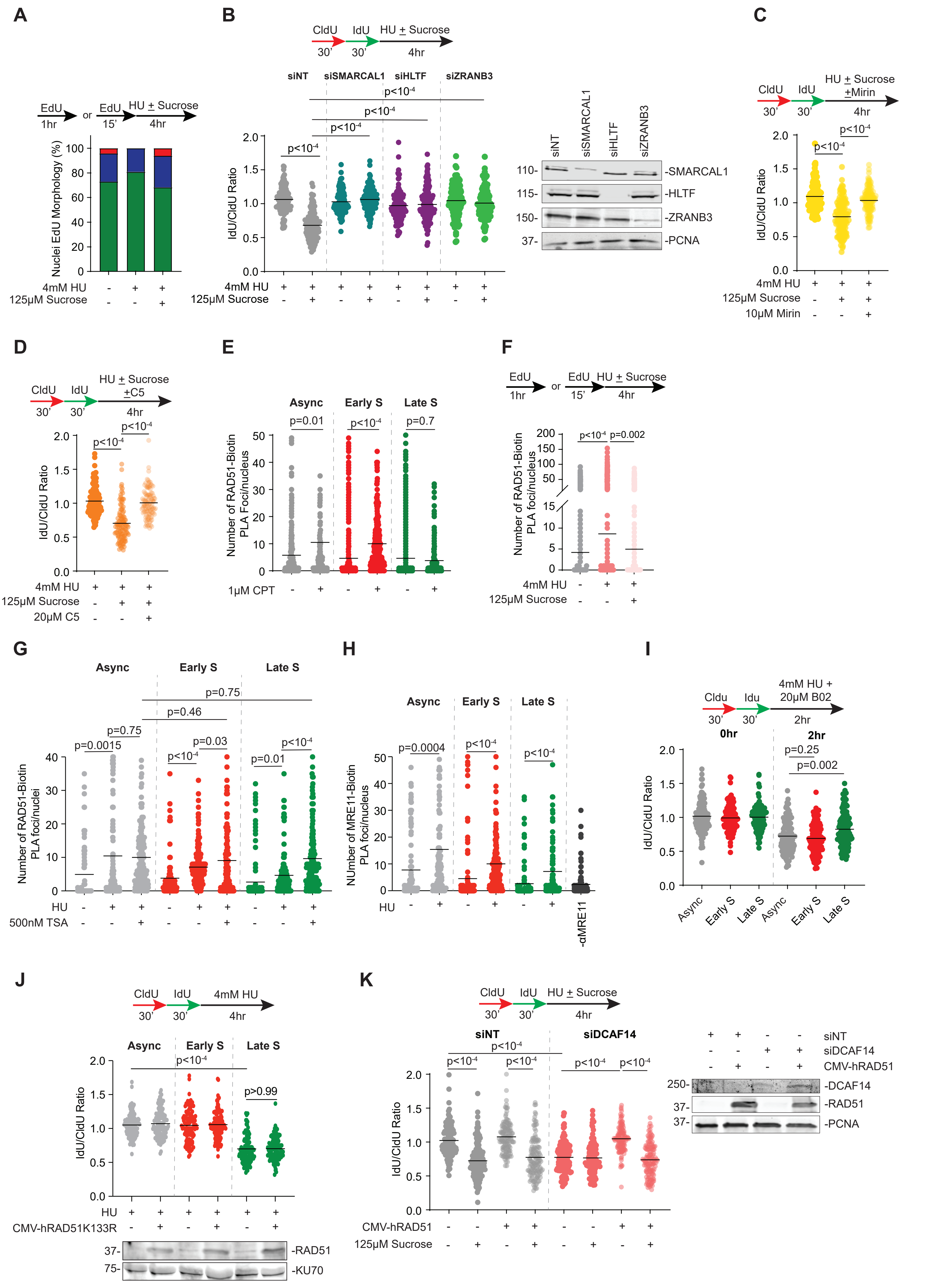

Supplementary Figure S4

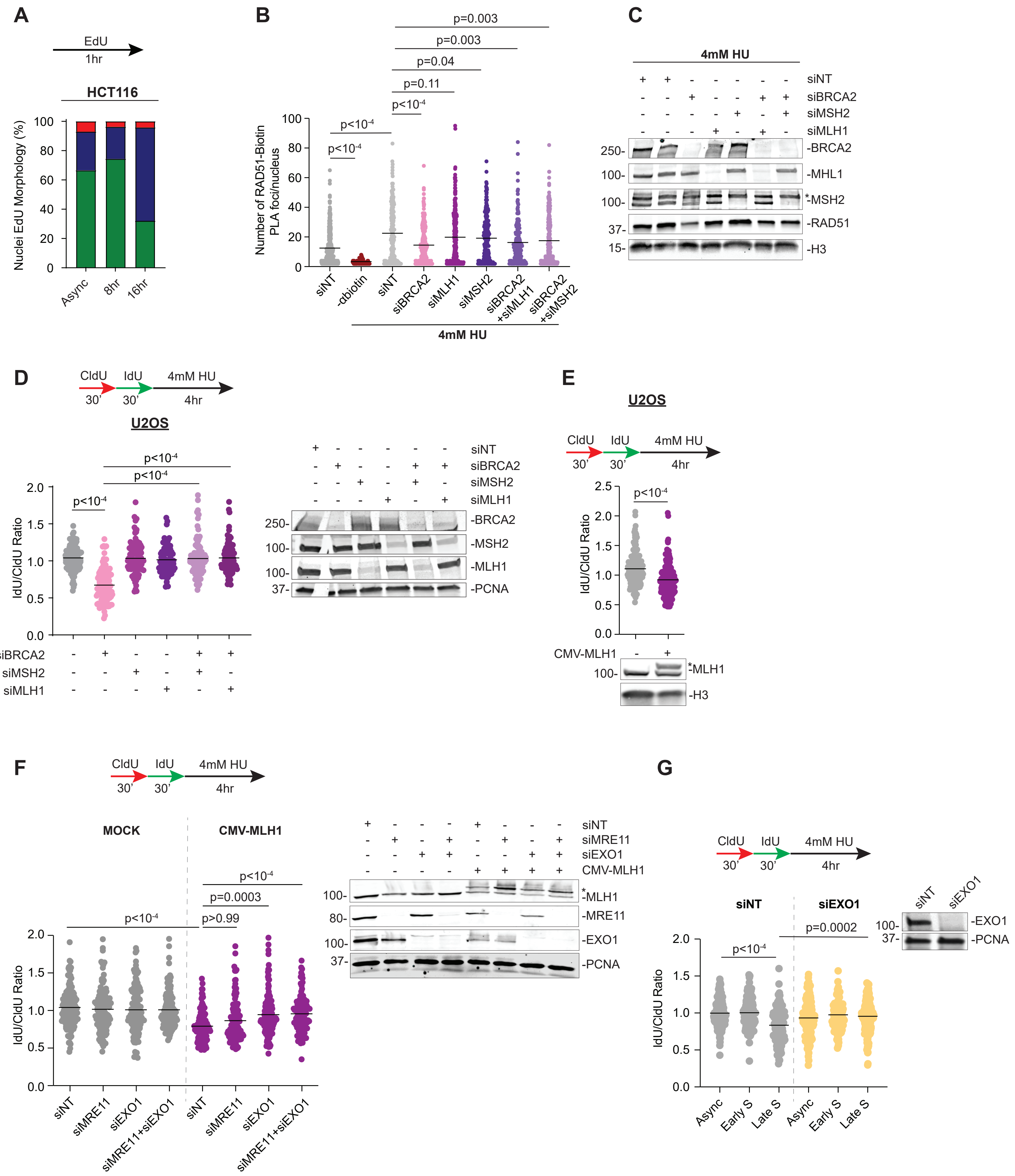

SUPPLEMENTARY TABLE AND FIGURE LEGENDS

Table S1. Representative EdU morphology distributions for experiments en masse. Table represents percent distributions of EdU morphology subtypes A, B, and C for the indicated figure panels. Numerical values from representative experiment are depicted.

Figure S1. (A) Representative immunofluorescence images depicting Async (left), Early S (middle) and Late S (right) RPE-1 cells stained for EdU (red), pH3.Ser10 (green) and DAPI (blue) are shown. Cells were either untreated or treated with HU for 4 hours. Graph represents mean $\pm$ S.E.M. (n>4). **(B)** Async (left panel), Early S (middle panel) and Late S RPE-1 (right panel) cells were harvested for cell cycle progression analyses using dual staining flow cytometry for EdU and DAPI. Percentages in black font represent populations in G1, S and G2/M. Percentages in green, blue and red font represent S-phase populations in early, mid and late S-phase respectively.

Figure S2. Fork stalling in late replication triggers nascent strand degradation. (A) Fork degradation assays were performed at 20 hours, 24 hours and 28 hours post serum starvation in RPE1-cells. **(B, C, D, E and F)** Fork degradation assays were performed in Async, Early S or Late S RPE-1 cells as indicated. In panel B, cells were treated with either 4mM HU, 0.4mM APH or 10mM APH for 4 hours as shown. In panel C, cells transfected with the indicated siRNAs were treated with HU for 4 hours as shown. Representative immunoblot is shown with PCNA as a loading control. In panel D, cells were treated either with HU alone or with HU and PARP inhibitor Olaparib for 4 hours as shown. In panel E, cells transfected with the indicated siRNAs were treated with HU for 4 hours as shown. Representative immunoblot is shown with PCNA as a loading control. * indicates non-specific band. In panel F, cells were treated with or without HU with simultaneous exposure to mirin and C5. Representative IdU/CldU ratios are plotted. **(G)** Fork recovery assays were performed in Async, Early S or Late S RPE-1 cells as indicated. Cells were treated either with HU alone or HU with mirin. Replication tracts with both IdU and CldU labels were quantified as percentage of all fibers analyzed. Graph represents mean $\pm$ S.E.M (n=3). p-values were derived using Kruskal-Wallis test with Dunn's multiple comparisons in panels A to F, and unpaired t tests in panel G. Black horizontal lines represent mean.

Figure S3. Chromatin compaction induces nascent strand degradation. (A) Bar graph represents percent (%) nuclei distribution for subtypes A (green), B (blue) and C (red) in Async RPE-1 cells treated as indicated. **(B)** Fork degradation assays were performed in RPE-1 cells transfected with the indicated siRNAs. Where indicated, assays were performed in presence of sucrose. Representative immunoblot is shown with PCNA as a loading control. **(C and D)** Fork degradation assays were performed in RPE-1 cells as indicated. **(E)** SIRF assays were performed in Async, Early S or Late S RPE-1 cells treated with or without CPT using antibodies targeting RAD51 and Biotin. **(F)** SIRF assays were performed in async RPE-1 cells treated with or without HU using antibodies targeting RAD51 and Biotin. Where indicated, cells were concomitantly treated with HU and sucrose. **(G)** SIRF assays were performed in Async, Early S or Late S RPE-1 cells treated with or without HU using antibodies targeting RAD51 and Biotin. Where indicated, cells were concomitantly treated with HU and TSA. **(H)** SIRF assays were performed in Async, Early S or Late S RPE-1 cells treated with or without HU using antibodies targeting MRE11 and Biotin. -αMRE11 represents sample with no MRE11 antibody. **(I)** Fork degradation assays were performed in Async, Early S or Late S RPE-1 cells as shown. **(J)** Fork degradation assays were performed in Async, Early S or Late S RPE-1 cells transfected with or without RAD51K133R cDNA. Immunoblot depicts RAD51 overexpression with KU70 as loading control. **(K)** RPE-1 cells transfected with the indicated siRNAs were co-transfected with or without RAD51 cDNA and subjected to fork degradation assays. Where indicated, assays were performed in presence of sucrose. Representative immunoblot is shown with PCNA as a loading control. p-values were derived using Kruskal-Wallis test with Dunn's multiple comparisons in panels (B), (C), (D), (I), (J) and (K), and unpaired t test in panels (E), (F), (G) and (H). Black horizontal lines represent mean.

Figure S4. MMR antagonizes BRCA2-RAD51 dependent stalled fork protection. (A) Bar graph represents percent (%) nuclei distribution for subtypes A (green), B (blue) and C (red) in Async, Early S or Late S HCT116 cells harvested post serum starvation at the indicated time points. **(B)** RPE-1 cells were treated as indicated and subjected to SIRF analysis using antibodies targeting RAD51 and Biotin. -αbiotin represents sample with no biotin antibody. **(C)** Lysates extracted from RPE-1 cells transfected with the indicated

siRNAs were probed with the antibodies as shown. Representative immunoblot is included with Histone H3 as loading control. * indicates non-specific band. **(D)** Fork degradation assays were performed in U2OS cells transfected with the indicated siRNAs. Representative immunoblot is shown with PCNA as loading control. **(E)** Fork degradation assays were performed in siRNA-transfected U2OS cells as indicated, with or without CMV-MLH1 cDNA co-transfection. Representative immunoblot is shown with Histone H3 as a loading control. * indicates epitope tagged MLH1. **(F)** Fork degradation assays were performed in mock or CMV-MLH1 transfected RPE-1 cells and co-transfected with the siRNAs as indicated. Representative immunoblot is shown with PCNA as loading control. * indicates epitope tagged MLH1. **(G)** Fork degradation assays were performed in Async, Early S or Late S RPE-1 cells as indicated. p-values were derived using Kruskal-Wallis test with Dunn's multiple comparisons in panels (D), (E), (F) and (G), and unpaired t test in panel (B). Black horizontal lines represent mean.
