# Supplementary material for: Replication fork stalling in late S-phase elicits nascent strand degradation by DNA mismatch repair
Source: Nucleic Acids Res. 2024 Aug 24;52(18):10999–1013. doi: 10.1093/nar/gkae721 (PMC11472054; doi:10.1093/nar/gkae721)
Supplement: gkae721_Supplemental_Files [file gkae721_supplemental_files.zip › Table S1 legends.docx]

Table S1. Representative EdU morphology distributions for experiments en masse. Table represents percent distributions of EdU morphology subtypes A, B, and C for the indicated figure panels. Numerical values from representative experiment are depicted.
